# Role of chloride on the instability of blue emitting mixed-halide perovskites

**DOI:** 10.1007/s12200-023-00088-x

**Published:** 2023-11-17

**Authors:** Max Karlsson, Jiajun Qin, Kaifeng Niu, Xiyu Luo, Johanna Rosen, Jonas Björk, Lian Duan, Weidong Xu, Feng Gao

**Affiliations:** 1 Department of Physics, Chemistry and Biology (IFM), Linköping University, Linköping, Sweden; 2 Key Lab of Organic Optoelectronics and Molecular Engineering of Ministry of Education, Department of Chemistry, Tsinghua University, Beijing, 100084 China; 3 Frontiers Science Center for Flexible Electronics, Xi’an Institute of Flexible Electronics (IFE), Northwestern Polytechnical University, Xi’an, 710072 China

**Keywords:** Ion migration, Blue electroluminescence, Mixed halide perovskites

## Abstract

Although perovskite light-emitting diodes (PeLEDs) have seen unprecedented development in device efficiency over the past decade, they suffer significantly from poor operational stability. This is especially true for blue PeLEDs, whose operational lifetime remains orders of magnitude behind their green and red counterparts. Here, we systematically investigate this efficiency-stability discrepancy in a series of green- to blue-emitting PeLEDs based on mixed Br/Cl-perovskites. We find that chloride incorporation, while having only a limited impact on efficiency, detrimentally affects device stability even in small amounts. Device lifetime drops exponentially with increasing Cl-content, accompanied by an increased rate of change in electrical properties during operation. We ascribe this phenomenon to an increased mobility of halogen ions in the mixed-halide lattice due to an increased chemically and structurally disordered landscape with reduced migration barriers. Our results indicate that the stability enhancement for PeLEDs might require different strategies from those used for improving efficiency.

**Graphical Abstract:**

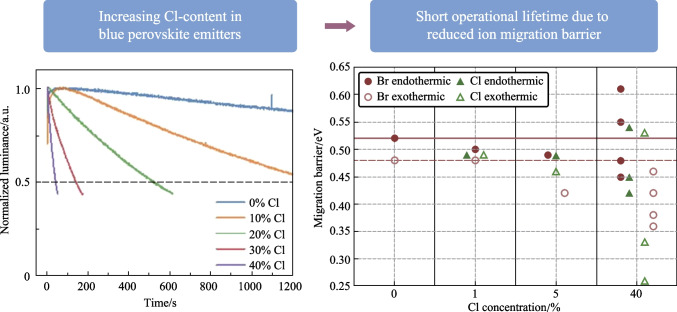

**Supplementary Information:**

The online version contains supplementary material available at 10.1007/s12200-023-00088-x.

## Introduction

While perovskite light-emitting diodes (PeLEDs) are making strides in approaching the standards of more established technologies like organic and quantum-dot light-emitting diodes (OLEDs and QLEDs) in external quantum efficiencies (EQEs), they are still far behind in operational stability [1]. This discrepancy can be attributed, in part, to the prevalent focus within the research community on efficiency optimization. Consequently, improvements in stability have often emerged as a side effect of EQE enhancements that stem from factors such as reduction of density of defect sites.

The quest for efficient devices has led to important insights, especially regarding the role of defects in metal-halide perovskites: how they relate to the optical quality of the material as well as how they can be suppressed through rational design of synthesis procedures [2–4]. Strategies to fabricate high-quality perovskite films include control over the stoichiometry [5–7], crystallization dynamics through, for example, different solvent strategies [8–10], as well as interfacial and post-growth treatments [11]. The use of additives is now also widely and successfully adopted (especially in PeLEDs) both to moderate film growth and to serve as passivating agents [12–15]. While diverse, most approaches share the underlying mechanism behind improved efficiency, by minimizing non-radiative losses through defect reduction.

There is a general consensus in the community that defects also play a negative role on the temporal stability of perovskite devices by facilitating ionic transport, acting as hopping sites for neighboring ions [16, 17]. Accompanied by increased optical quality of the perovskite film through defect reduction, an increase in device stability was typically also reported [6, 7, 14, 15]. However, state-of-the-art PeLEDs often use perovskite emitters with quantum yields approaching unity, limiting the efficiency of the best emitters mainly by poor light outcoupling [18]. Hence, defects alone can hardly account for the poor device lifetimes fully. This is especially true for blue-emitting perovskites, which are not only less efficient than their green and red counterparts but are orders of magnitude behind regarding operational stability [1].

Here, we conducted a systematic study on the role of Cl-incorporation (necessary for blue emission in three-dimensional perovskites) on device stability and EQE. Employing the most straightforward method of bandgap tuning in perovskites by adjusting the halide ratio through mixing chloride and bromide anions, we fabricated a series of PeLEDs emitting in the spectral region from green to blue. A direct correlation between blue-shifting of the emission by Cl-incorporation and reduced operational lifetime is found. Interestingly, even though Cl does not have a large effect on the EQE at moderate Cl-incorporation (≤ 30%), even small amounts of Cl (5%) greatly decrease the device’s half-lifetime (${t}_{50}$). A negative exponential relationship between ${t}_{50}$ and Cl-content is found as well as increasing dynamical rate of change in electrical properties of the devices during operation. We assign this to the increased mobility of halogen ions in the mixed halide lattice due to an increased chemically and structurally disordered landscape with reduced migration barriers. Under the applied electrical field, ions readily move across the device, resulting in large injection barriers and degraded electrical properties. The discrepancy between the EQE and ${t}_{50}$ trends further paints the picture that the problem of enhancing device stability is not necessarily solved by the approaches used to maximize device efficiency, due to different underlying mechanisms, and hence requires alternative solutions.

## Results and discussion

**Fig. 1:**
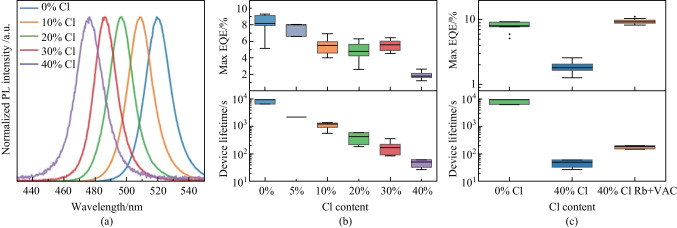
**a** Normalized photoluminescence spectra from mixed-halide perovskite films. **b** Statistics for maximum EQE and operational lifetime (${t}_{50}$) at different halide ratios. **c** Statistics for maximum EQE and operational lifetime at 0% and 40% Cl with and without Rb and vapor-assisted crystallization

A series of PeLEDs were fabricated with the emissive material based on our previous work on blue-emitting mixed-halide perovskites (details in the experimental section) [9]. In brief, precursors with a stoichiometry of Cs:FA:Pb:[Br_1−__*x*_ + Cl_*x*_] (1.2:0.3:1:3.5) were dissolved in dimethyl sulfoxide (DMSO), where the chloride content, $x$, was varied between 0% and 40% to tune the emission from green to blue. A device architecture consisting of indium tin oxide (ITO)/nickel oxide (NiO_*x*_)/poly(9-vinylcarbazole) (PVK)/polyvinylpyridine (PVP)/perovskite/2,2′,2″-(1,3,5-benzinetriyl)tris(1-phenyl-1*H*-benzimidazole) (TPBi)/lithium fluoride/aluminum, was used. PeLED devices were unencapsulated and tested in ${\mathrm{N}}_{2}$-atmosphere inside a glovebox. As visible from photoluminescence measurements in Fig. 1a, the optical bandgap of the perovskite displays a close relationship with respect to stoichiometric tuning of the halide content in the precursor solution. Photoluminescence (PL) peaks are centered at approximately 520 and 478 nm for compositions with 0% Cl and 40% Cl, respectively.

Figure 1b summarizes device statistics of maximum EQE (EQE_max_) from current density–voltage (*J–V*) sweeps and operational lifetime measurements, ${t}_{50}$ (defined as a degradation to 50% of the initial luminance) at different levels of Cl-incorporation. *J–V* curves were measured by sweeping the voltage with steps of 0.1 V/s, and device lifetimes were probed by applying a constant current density resulting in an initial luminance of approximately 100 $\mathrm{cd}/{\mathrm{m}}^{2}$. Across the range of investigated devices, the current density and initially applied voltage required varied in the range of 0.7–1.5 $\mathrm{mA}/{\mathrm{cm}}^{2}$ and 2.9–3.2 V, respectively (except for the 40% Cl which required a much larger current density and bias voltage of 13 $\mathrm{mA}/{\mathrm{cm}}^{2}$ and 3.9 V). Luminance *vs* voltage, *J–V* curves and EQE *vs* current density for representative devices are shown in Fig. S1.

Notably, EQE only slightly drops upon moderate Cl-addition,  ≤ 30%, while the ${t}_{50}$ decreases exponentially, from an average of 2.5 h for the pure Br-device to a mere 110 s for the 30% Cl-devices, as seen in Fig. 1b. It is also clear that even a small amount of Cl (5%) has a large negative impact on the device stability, while showing no significant drop in EQE. Hence, we see no straightforward relationship between device EQE and stability of these mixed-halide PeLEDs, which contrasts with previous ideas about the correlation between perovskite device efficiency and operational lifetime [19].

The emission properties of perovskite films are typically considered to be indicative of the intrinsic defect properties [3]. Therefore, we used steady-state PL and time-correlated single-photon counting to probe the optical quality of our films qualitatively (Fig. S2). A drop in both PL intensity and PL lifetime is seen when increasing the Cl content in the perovskite films, suggesting an increased defect density resulting in trap-mediated non-radiative losses. This trend follows the EQE behavior with respect to Cl-incorporation but cannot itself explain the strong reduction in device lifetime in the mixed-halide films.

To further examine the discrepancy between EQE and lifetime, we investigated films and devices at a fixed Cl-content of 40%, while adopting our previously developed method of vapor assisted crystallization (VAC) and rubidium-incorporation to improve the quality of the perovskite film [9] (Supplementary Note 1). As visible from the almost ten-fold increase in PLQY and a large increase in PL-lifetime, as well as an enhancement of device EQE to almost 12% at 40% Cl (from ~ 1% for the control films), the material quality was evidently improved (Fig. 1; Fig. S3). Surprisingly, even though the device efficiency was drastically improved, the operational lifetime was maintained at a few minutes, still orders of magnitudes shorter than that of the pure Br device (Fig. 1c). This is surprising as the Cl-free film displayed both lower EQE, as well as shorter carrier lifetimes than the Cl-containing VAC/Rb-film.

Cl-vacancies are known to create trap states with deeper energy levels than those of their Br- and I-counterparts [20–22]. Consequently, a significant increase in Cl-vacancies would be expected to have a notable impact on both PL and EQE. However, such an effect is not evident in our study. This leads us to conclude that inherent imperfections within the bulk of the perovskite are likely not the primary factor contributing to the diminished operational stability of our chlorine-containing PeLEDs when compared to the behavior of their pure bromide counterparts. Furthermore, we note that the morphologies of the pure Br films and the VAC/Rb-films display a high resemblance (Fig. S4), both consisting of discontinuous networks of large grains. Hence, we also exclude the impact of film morphology on the difference in the lifetime of the devices.

**Fig. 2:**
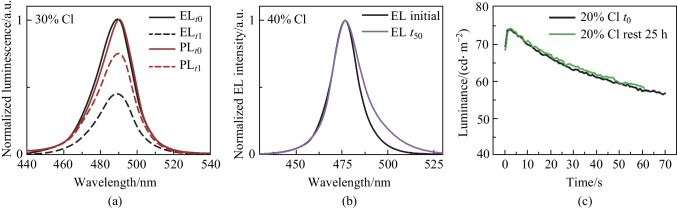
**a** Initial electroluminescence and at ${t}_{50}$ together with photoluminescence before and after running the device. **b** Spectral shift during constant current test for 40% Cl-content device. **c** 20% Cl device tested to 80% degradation followed by rest for 25 h and a new test

To further discern between the optical degradation of the perovskite layer from other factors during operation, we measured the PL/EL intensity before (EL_*t*0_ and PL_*t*0_) and after operation (EL_*t*1_ and PL_*t*1_) to end of lifetime, ${t}_{50}$ (Fig. 2a). Notably, the half-degraded devices still retained ~ 75% of the initial PL-intensity. If the decrease in light output had originated from the perovskite thin films, one would have expected a similar drop in PL intensity. From this, we draw the conclusion that the device degradation is not mainly determined by the optical quality of the bulk perovskites, but rather likely by effects at the interfaces or in adjacent layers [23, 24]. A slight redshift of the emission peak accompanied by a spectral broadening towards longer wavelengths was seen after operation for the mixed halide perovskites with a large Cl-content (Fig. 2b; Fig. S5). This indicates a bromide-enrichment in the recombination zone of the emitter, and further, ions were moving and rearranging under electrical bias. Additionally, upon driving the devices to a moderate degradation of ${t}_{80}$ we saw a reversible effect upon resting (Fig. 2c; Fig. S6), suggesting that at limited device degradation, the effects are also reversible and dynamic in nature. This further indicates that the device dynamics, at low to moderate degradation, are mainly driven by mobile species in the device that diffuse back after bias has been removed, as previously reported [24].

**Fig. 3:**
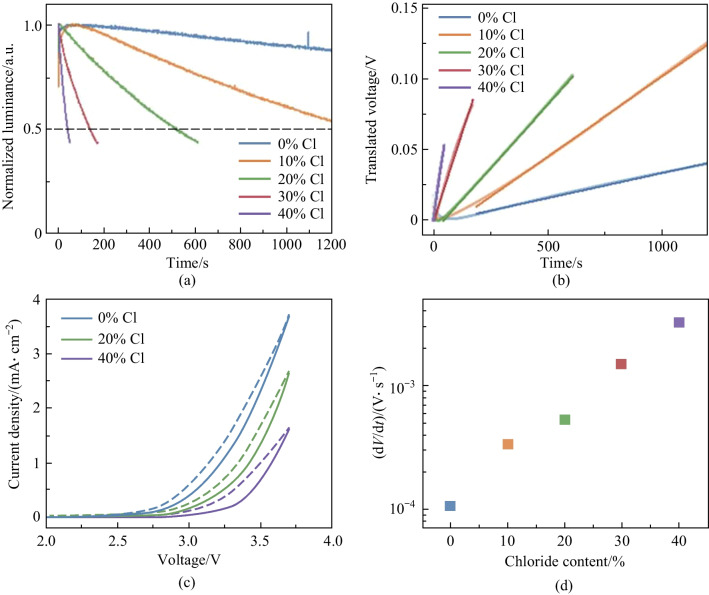
**a** Normalized luminance as a function of time at constant current bias ($0\% :0.7\, \mathrm{mA}/{\mathrm{cm}}^{2},10{\% }:1.2\,\mathrm{ mA}/{\mathrm{cm}}^{2},20\,{\% }:0.7\,\mathrm{ mA}/{\mathrm{cm}}^{2},30{\% }:1.5\,\mathrm{ mA}/{\mathrm{cm}}^{2},40\,{\% }:13.0\,\mathrm{ mA}/{\mathrm{cm}}^{2})$. **b** change in applied voltage needed to sustain a constant current density. Curves translated to align to the voltage minima (original data in Fig. S8). **c** Voltage sweeps at different Cl-incorporation. **d** Rate of change in voltage from fitting the slow rise in voltage in Fig. 3b

We continued by investigating the electrical characteristics of PeLEDs during operation. The luminance as a function of time at a fixed current density (Fig. 3a; Fig. S7), displayed an initial rise upon biasing the device followed by a slower decrease in light output. A similar but opposing trend was observed for the voltage characteristics of the device during the same time span (Fig. 3b; Fig. S7) The voltage needed to sustain the current density was dynamically changing with time, displaying an initial fast drop (Fig. S7) followed by a slow increase, mirroring the luminance behavior. We also note that the point in time of maximum luminance overlaps with the lowest driving voltage, indicating that these phenomena are closely linked and likely share underlying mechanisms.

We found that the rate at which the luminance (voltage) is changing is highly dependent on perovskite stoichiometry, where both the initial rise (drop) and subsequent decay (rise) are faster as more Cl is incorporated into the perovskite. This is also true for device hysteresis (typically associated with halogen migration in both perovskite solar cells and LED), which becomes more pronounced with Cl-incorporation (Fig. 3c) [25, 26]. We fit the rate of change of applied voltage as a function of time, d*V*/d*t*, using a simple linear function after the voltage minimum, as shown in Fig. 3b, with resulting values listed in Table S1. These values exhibit an exponential relationship between d*V*/d*t* and halide ratio (Fig. 3d), coinciding with the exponential drop in device lifetime for the same perovskites (Fig. 1b*).*

**Fig. 4:**
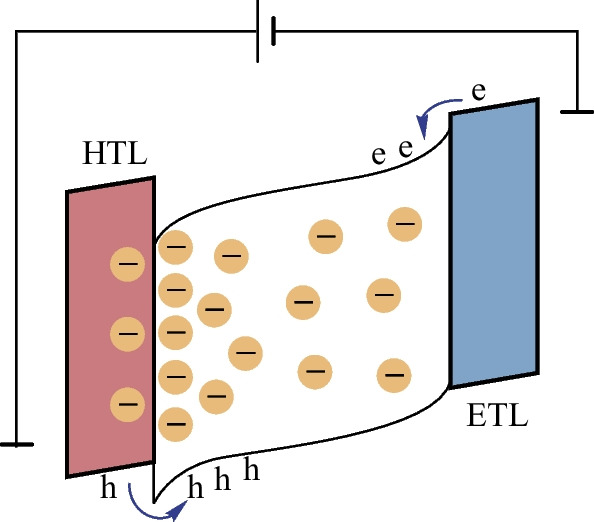
Illustration of the proposed electrical field-induced ion migration and accumulation of ions, leading to field screening, band bending and increased injection barrier. Here HTL and ETL denote hole transport and electron transport layers, respectively

We rationalize this behavior through the understanding of mobile ionic species in the perovskite film and their redistribution under an applied electrical field [27], illustrated in Fig. 4. Upon bias, an initial moderate accumulation of charged species close to interfaces can cause band bending due to local doping and/or give rise to interfacial dipoles, leading to reduced injection barriers and improved charge balance [28]. This can be beneficial for efficient and balanced carrier injection [29, 30] and is visible through the initial reduction in voltage needed to sustain a fixed current density as well as the increased luminance. Over time, ion redistribution and aggregation can alter the built-in electrical field across the device, leading to field screening and increased injection barriers, significantly affecting carrier injection. Prolonged bias likely causes immobilized ions and electrochemical reactions at interfaces, in transport layers and at contacts, leading to irreversible changes [31, 32].

As we see a clear relationship between halogen-mixing and dynamical processes in our devices, we carried out first-principles density functional theory (DFT) calculations to assess kinetics of migration behaviors of halogens in the $\mathrm{CsPb}{\left({\mathrm{Br}}_{1-x}{\mathrm{Cl}}_{x}\right)}_{3}$. The energy barriers for halogen migration are obtained by calculating transition state energies for halogens drifting from their original site to the closest vacancy. It is noted that the energy difference between the initial state (IS) and final state (FS) of migration may not be 0 eV as the energies of IS and FS are dependent on the site of vacancy. Therefore, barriers are considered from both sides of the migration path, firstly, the energy barrier of exothermic path (${E}_{\mathrm{A}}^{\mathrm{exo}}$), in which the IS exhibits higher energy than the FS, and secondly, the energy barrier of the endothermic path, with the energy of IS lower than the FS. More details about the DFT calculations are summarized in Sect. 4.

**Fig. 5:**
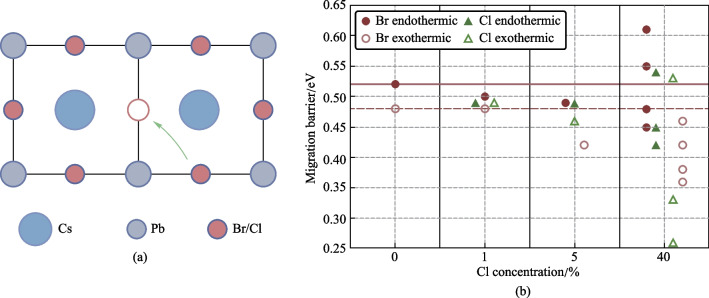
**a** Vacancy mediated ion-migration along the equatorial path. **b** Calculated migration barriers for Br (brown) and Cl (green) ions

Figure 5 and Table 1 summarize the calculated migration barriers of halogens in the CsPb(Br_1−*x*_Cl_*x*_)_3_ with Cl concentration from 0% to 40%. Overall, the migration barriers for halogen diffusion display a broad distribution, highly dependent on the local initial and final chemical environment of the migrating ions. The Br migration in the CsPbBr_3_ exhibits an energy barrier of 0.48 eV, which is in good agreement with the previous study [33]. In addition, migration barriers of Br and Cl remain at similar values when the Cl concentration is 1% (only 1 Cl atom in the supercell). As the Cl concentration increases to 5%, a decrease in migration barriers can be found for both Br and Cl, indicating the possibility for increases of halogen migration. In the case of 40% Cl perovskites, the migration of halogens becomes highly dependent on the local chemical environment. This situation prohibits the migration of certain halogens. Nevertheless, the major kinds of mobile halogens are subject to substantial drops in their migration energy barriers, compared to observed behavior with pure Br and low Cl concentration (1%). The size of these decreases reaches as much as ~ 0.12 and ~ 0.23 eV for Br and Cl anions, respectively. The reduced barriers lead to the increased probability of ion-hopping (*P*), according to Eyring equation ($P \propto {\text{e}}^{\frac{E_{\text{A}}}{k_{\text{B}}T}}$). Such results are therefore in agreement with the experimental results of an increased rate of change in electrical properties, rationalized through ionic movement and resulting effects.

**Table 1:** Calculated migration barriers for Br and Cl in the CsPb(Br_1−*x*_Cl_*x*_)_3_

Cl concentration/%	${E}_{\mathrm{A}}^{\mathrm{exo}}(\mathrm{Br})$/eV	${E}_{\mathrm{A}}^{\mathrm{endo}}(\mathrm{Br})$/eV	${E}_{\mathrm{A}}^{\mathrm{exo}}(\mathrm{Cl})$/eV	${E}_{\mathrm{A}}^{\mathrm{endo}}(\mathrm{Cl})$/eV
**0**	0.48	0.52	–	–
**1**	0.48	0.50	0.49	0.49
**5**	0.42	0.49	0.46	0.49
**40**	0.38	0.55	0.26	0.42
	0.42	0.45	0.53	0.54
	0.46	0.61	0.33	0.45
	0.36	0.48	–	–

## Conclusion

To conclude, we have examined the discrepancy between device efficiency and operational lifetime in PeLEDs by investigating a series of mixed-halide perovskites emitting from the green to blue region. We have shown that the underlying mechanism responsible for device degradation during operation is different from that deciding efficiency, as seen from the large discrepancy between ${t}_{50}$-values across the devices and EQEs. While EQE is sensitive to optically active defects in the perovskite layer leading to non-radiative recombination, t_50_ is more closely related to the mobile ionic species. The reduced barriers for halogen migration in the mixed Br/Cl-system result in a rapid degradation of the electrical properties of our PeLED-devices. As such, the problem of poor operational stability of PeLEDs needs to be addressed as its own separate problem and with different methods from those used for device efficiency improvements. We speculate that the solution will likely be found in ways to immobilize the ionic species in the perovskite films as well as to further prevent ions moving into the adjacent layers and contacts.

## Experimental methods

### Materials

Caesium bromide (CsBr, 99.9%), lead bromide (PbBr_2_, 99.999%), lead chloride (PbCl_2_, ultradry beads, 99.999%) were purchased from Alfa Aesar. Formamidinium bromide (FABr) was purchased from Greatcell Solar. Polyvinylpyridine (PVP, average Mw ~ 55,000), 4,7,10-trioxa-1,13-tridecanediamin (TTDDA), rubidium bromide (RbBr, 99.99%), and poly(9-vinylcarbazole) (PVK, average Mn 1,100,000) were purchased from Sigma Aldrich. NiO_*x*_ nanocrystals dispersed in ethanol were purchased from Avantama AG. 1,3,5-tris(1-phenyl-1*H*-benzimidazol-2-yl)benzene (TPBi) was purchased from Luminescence Technology corp. Other materials for device fabrication were all purchased from Sigma-Aldrich.

### Perovskite precursor

Perovskite precursors (CsBr:FABr:PbBr_2_:PbCl_2_:TTDDA) with a molar ratio of (1.2:0.3:*x*:*y*:0.1) where (*x* + *y* = 1) were mixed and dissolved in dimethyl sulfoxide (DMSO). For the Rb-containing solutions, some FA was replaced by Rb to create a precursor solution of (CsBr:FABr:RbBr:PbBr_2_:PbCl:TTDDA) (1.2:0.2:0.1:*x*:*y*:0.1). The precursor concentration as determined by Pb^2+^ was 0.15 mol/L. The precursor solutions were stirred at 80 °C for 2 h before use.

### PeLED fabrication

Prepatterned indium tin oxide (ITO) films on glass were sequentially cleaned by detergent, TL-1 (a mixture of water, ammonia (25%) and hydrogen peroxide (28%) (5:1:1) by volume), and UV-ozone for 15 min. NiO_*x*_-solution was spin-coated in air at 4000 r/min for 30 s and subsequently annealed at 140 $^\circ{\rm C}$ for 15 min. The substrates were then transferred into a glovebox (< 0.1 ppm water, < 1 ppm oxygen). PVK (4 mg/mL in chlorobenzene) was deposited at 3000 r/min for 30 s and annealed at 120 $^\circ{\rm C}$ for 10 min. PVP (3 mg/mL in isopropyl alcohol) was deposited at 3000 r/min and annealed at 100 $^\circ{\rm C}$ for 5 min. The perovskite solution was deposited at 3000 r/min for 30 s and annealed at 80 $^\circ{\rm C}$ for 10 min. For the VAC-films, directly after spin-coating, the films were put in a ⌀60 mm petri-dish (with lid) containing 20 µL DMF and kept there for 20 min when they were subsequently annealed at 80 $^\circ{\rm C}$ for 10 min. Finally, the electron transport layer TPBi and top contacts LiF/Al (1 nm/120 nm) were deposited by thermal evaporation through shadow masks at a base pressure of ~ 10^−^^7^ torr. The device area was 7.25 ${\mathrm{mm}}^{2}$. 

### PeLED characterization

All PeLED device characterizations were performed at room temperature in a nitrogen filled glovebox without encapsulation. A Keithley 2400 source-meter and a fiber connected integration sphere (FOIS-1) coupled with a QE Pro spectrometer (Ocean Optics) were utilized. The absolute radiance was calibrated by a standard Vis–NIR light source (HL-3P-INT-CAL plus, Ocean Optics). The PeLED devices were measured on top of the integration sphere and only forward light emission was collected. The devices were swept from zero bias to forward bias with a step voltage of 0.1 V, with 100 ms at each voltage step for stabilization. The device lifetime measurements were conducted using the same system.

### Film characterization

Steady-state PL spectra analyses of the perovskite films were carried out by a 405 nm laser as the excitation source. Emitted light was collected using a system of lenses directing the light to an optical fiber coupled to a Shamrock 303i spectrograph. Time-correlated single photon counting (TCSPC) measurements were carried out by using an Edinburgh Instruments FL1000 with a 405 nm pulsed picosecond laser (EPL-405). Top-view scanning electron microscope (SEM) images were acquired using LEO 1550 Gemini.

### Density functional theory (DFT) calculations

All the DFT calculations were performed by Vienna ab-initio simulation package (VASP) [34]. The projector augmented wave (PAW) method was employed to describe the electron–ion interactions, together with a plane wave basis expanded to a cutoff energy of 400 eV [35]. The exchange–correlation interactions were treated by the van der Waals density functional with the version of rev-vdWDF2 [36, 37]. The cubic crystal structure of CsPbX_3_, corresponding to space group P*m3m*, was employed to model the perovskite structure. The lattice constant of the CsPbBr_3_ was optimized to 5.89 Å, agreeing well with the previous theoretical study (5.87 Å) [33]. The structure of CsPb(Br_1−*x*_Cl_*x*_)_3_ was modelled by a 4 × 4 × 2 supercell, which consisted of 32 Cs, 32 Pb, and 96 halogen atoms (Br and Cl). The halogen atoms were randomly distributed in the supercell. The lattice constants of CsPbBr_3−*x*_Cl_*x*_ supercells were optimized to obtain their ground states. The halide vacancies were modeled by removing one halogen in the supercell, resulting in a vacancy concentration of 1/96 ≈ 1.04%. The effect of the halogen vacancy on the lattice constant was neglected due to the low vacancy concentration. The migration barriers of halogens were calculated by a combination of the climb image nudged elastic band (CI-NEB) method and the Dimer method [38, 39]. Firstly, the 10 images were generated between the initial and final states. The central image was then used as the input of the Dimer method to obtain accurate transition state energy. A 16 × 16 × 16 grid was employed to model the Brillouin zone to optimize the lattice constant, and a 2 × 2 × 1 grid was used for calculations of supercells. All structures and saddle points were optimized until the force acting on all atoms was below 0.01 eV/Å.

### Supplementary Information

Below is the link to the electronic supplementary material.

Supplementary file1 (PDF 641 KB)

## Data Availability

The data that support the findings of this study are available from the corresponding author, upon reasonable request.
