# Supplementary material for: Role of chloride on the instability of blue emitting mixed-halide perovskites
Source: Front Optoelectron. 2023 Nov 17;16(1):37. doi: 10.1007/s12200-023-00088-x (PMC10656409; doi:10.1007/s12200-023-00088-x)
Supplement: Supplementary file 1 — Supplementary file1 (PDF 641 KB) [file 12200_2023_88_MOESM1_ESM.pdf]

Supporting information for

Role of Chloride on the Instability of Blue Emitting Mixed-Halide Perovskites

Max Karlsson[1], Jiajun Qin[1], Kaifeng Niu[1], Xiyu Luo[1,2], Johanna Rosén[1], Jonas Björk[1], Lian Duan[2], Weidong Xu[1,3], Feng Gao[1*]*

[1]Department of Physics, Chemistry and Biology (IFM), Linköping University, Linköping, Sweden

[2]Key Lab of Organic Optoelectronics and Molecular Engineering of Ministry of Education, Department of Chemistry, Tsinghua University, Beijing 100084, China

[3]Frontiers Science Center for Flexible Electronics, Xi'an Institute of Flexible Electronics (IFE), Northwestern Polytechnical University, 127 West Youyi Road, Xi'an, 710072, China

Supplementary Note 1:

Vapour-assisted crystallization. Our previously reported method of vapor-assisted crystallization is described here in short. Briefly, the as-spun films are directly put into ⌀60 mm petri-dish (with lid) where 20 µl DMF had been put inside 10 minutes prior. This creates an environment rich in solvent vapor which allows the perovskite film to stay in the liquid phase for a longer time, prolonging and retarding the crystallization process (or to some extent also allows for recrystallization). The solvent vapor provides a diffusive environment where initial compositional inhomogeneities can rearrange and equilibrate, driven by the chemical potential difference between Cl-rich and Br-rich regions. This has the resulting effect of homogenizing the film composition, as well as creating larger grains through Ostwald ripening processes and reducing film defects. For a more thorough description, we refer to our previous paper [1].

Supplementary Figures:

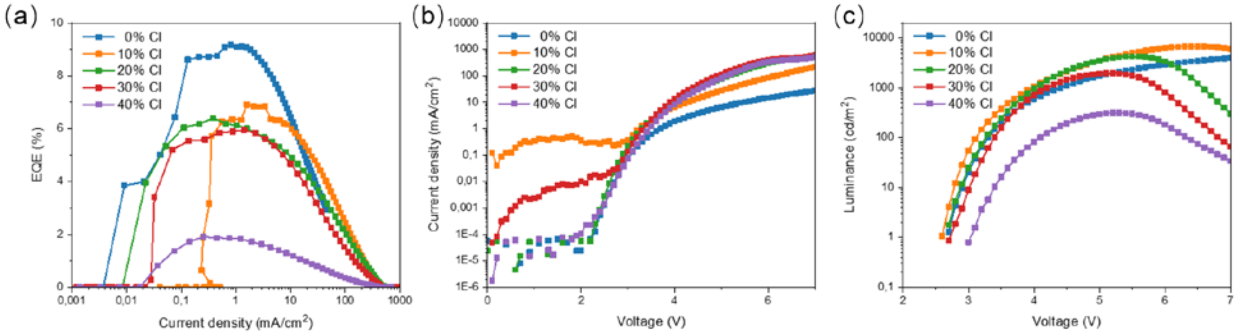

Fig. S1 Representative device characteristics at different levels of Cl incorporation. a, EQE-Current density (EQE-J). **b**, Current density-voltage (J-V). **c**, Luminance- voltage (L-V).

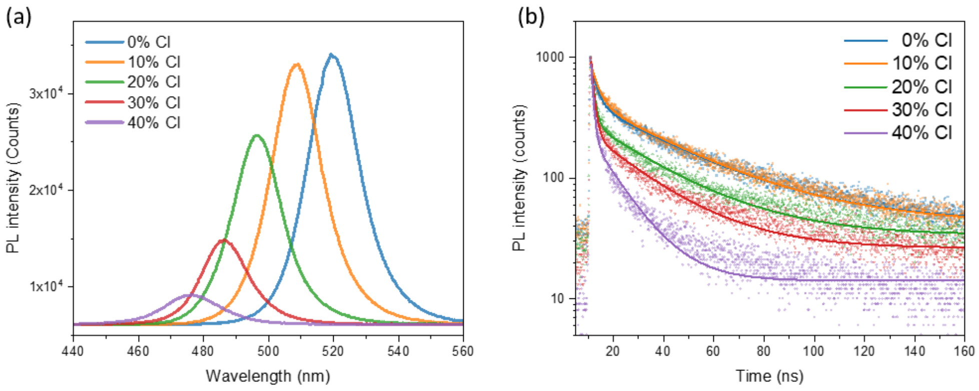

Fig. S2 **a**, photoluminescence and **b**, Time-resolved photoluminescence for perovskites with various amounts of Cl content.

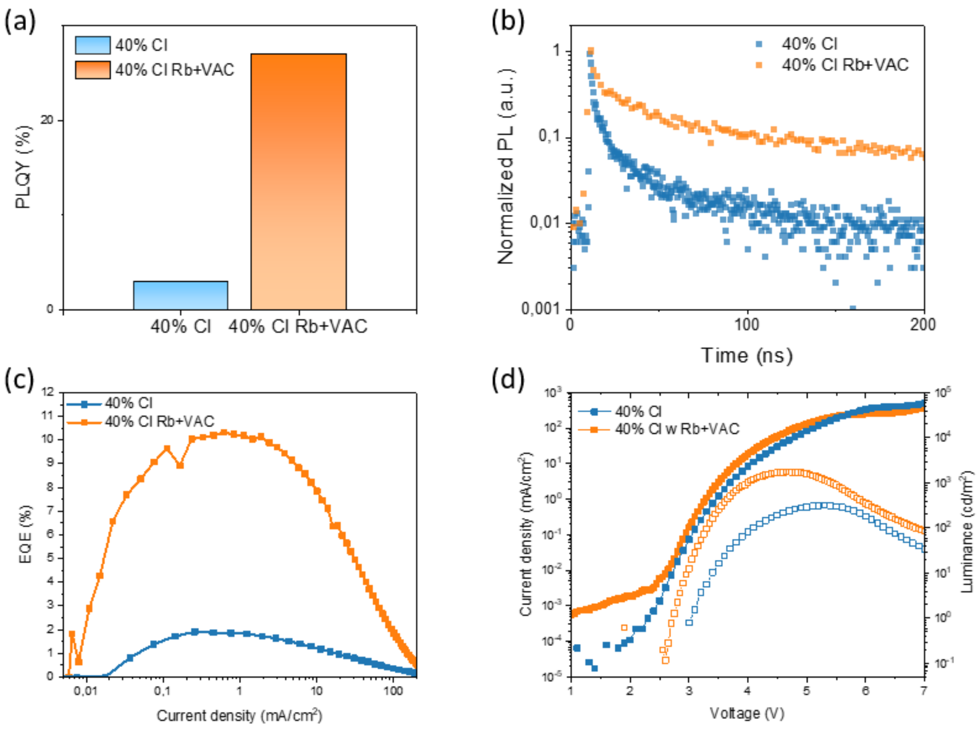

Fig. S3 **a**, PLQY of films with and without Rb addition and VAC **b**, Time-resolved photoluminescence of films with and without Rb and VAC. **c**, Current density-EQE curves. **d**, Current density and luminance as a function of applied voltage.

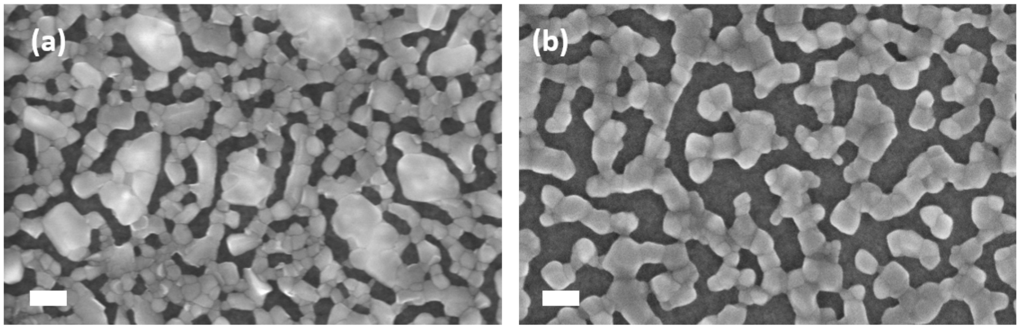

Fig. S4 SEM surface micrographs of **a**, 0% Cl, **b**, 40% Cl VAC/Rb. Scale bars are 200 μm.

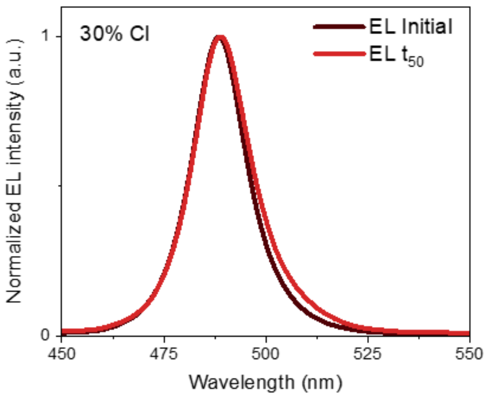

Fig. S5 Normalized EL spectra at the beginning of constant current measurement and at t_{50}.

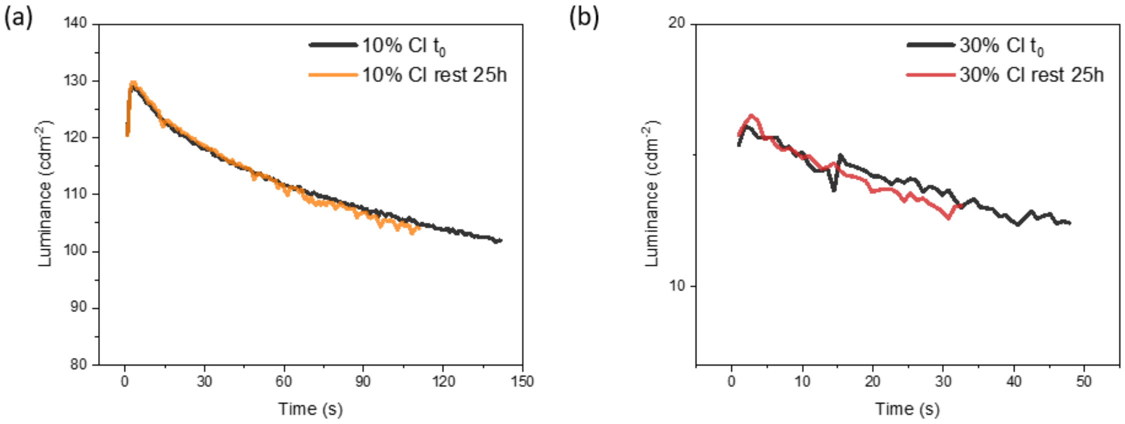

Fig. S6 Devices tested to 80% degradation and tested followed by a rest for 25h and 20h, respectively. **a**, For perovskites with 10% Cl content. b, For perovskites with 30% Cl content.

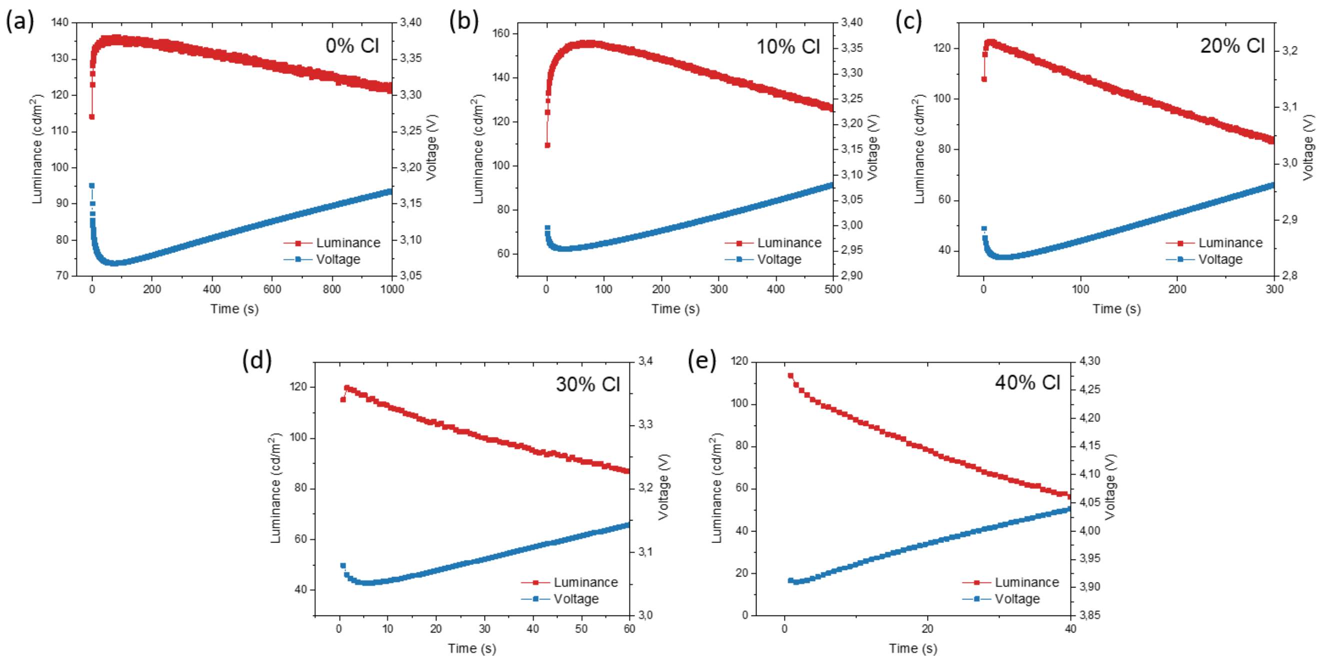

Fig. S7 Luminance and voltage characteristics as a function of time at constant current measurements at different Cl-contents.

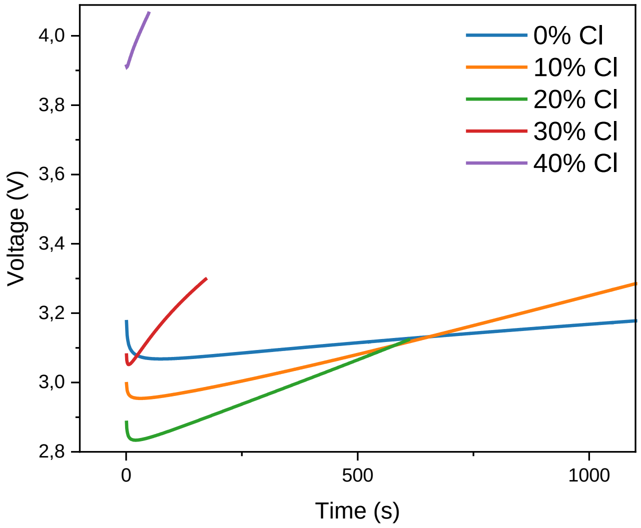

Fig. S8 Change in applied voltage needed to sustain a constant current density for different Cl-contents

Table S1: Voltage rate of change for different Cl-contents from fitting the slope in figure 3b in the main text.

**Chloride content (%)**	**dV/dt (Vs^{-1})**
0	1.05×10^{-4}
10	3.32×10^{-4}
20	5.27×10^{-4}
30	1.47×10^{-3}
40	3.26×10^{-3}

References

1. Karlsson, M., Yi, Z., Reichert, S., Luo, X., Lin, W., Zhang, Z., Bao, C., Zhang, R., Bai, S., Zheng, G., Teng, P., Duan, L., Lu, Y., Zheng, K., Pullerits, T., Deibel, C., Xu, W., Friend, R., Gao, F.: Mixed halide perovskites for spectrally stable and high-efficiency blue light-emitting diodes. Nat. Commun. **12**(1), 361 (2021).
